# The Long-COVID Experience Changed People’s Vaccine Hesitancy but Not Their Vaccination Fear

**DOI:** 10.3390/ijerph192114550

**Published:** 2022-11-05

**Authors:** Mirko Duradoni, Mustafa Can Gursesli, Letizia Materassi, Elena Serritella, Andrea Guazzini

**Affiliations:** 1 Department of Education, Literatures, Intercultural Studies, Languages and Psychology, University of Florence, 50135 Florence, Italy; 2 Department of Information Engineering, University of Florence, 50139 Florence, Italy; 3 Department of Social and Political Sciences, University of Florence, 50127 Florence, Italy

**Keywords:** vaccine hesitancy, vaccination fear, long-COVID, COVID-19

## Abstract

Starting in early 2020, the COVID-19 pandemic has been responsible, worldwide, for millions of deaths and patients with long-COVID syndrome. In an attempt to stop the spread of the virus, the blanket administration of COVID-19 vaccines proved to be the most effective measure, yet the existence and availability of functional vaccines did not and, still, do not ensure the willingness and intent of people to be vaccinated. This study assessed the similarities and differences in vaccine fears and vaccine hesitancy through between clusters of subjects: people that were not infected with COVID-19, people that had COVID but did not develop long-lasting symptoms, and people that were infected with COVID and developed long-COVID syndrome. From the sample of 1111 Italian people, it was found that individuals who experienced mild symptoms showed higher vaccine hesitancy (confidence, complacency, and collective responsibility) than those who did not contract COVID-19. People affected by long-COVID showed a lower overall hesitancy than individuals who had COVID-19 without incurring long-lasting symptoms and, thus, essentially resembled people who had no experience of COVID-19 infection in terms of the vaccine hesitancy scores. Vaccine fear remained unchanged across all three of the examined clusters.

## 1. Introduction

SARS-CoV-2 was discovered in Wuhan in late 2019 [1] and, since then, the new coronavirus has spread all over the world, leading the WHO to declare the COVID-19 outbreak a global pandemic on 11 March 2020 [2].

Currently (July 2022), the COVID-19 pandemic, worldwide, has led to 567,000,000 confirmed cases and 6,380,000 deaths [3]. The regions in the most difficult situations are Europe (239,000,000 cases, about 42% of the total cases of contagion) and the Americas (with 168,000,000 cases, approximately 30% of the total cases), while Africa is at the bottom of the list of regions, with around 9 million confirmed cases (1.5% of the global infections). It should be noted that these data refer only to the WHO-verified infections, and thus the “real” COVID-19 rates could reasonably be higher than those recorded due, for example, to asymptomatic patients, COVID-19 negationists who do not test themselves [4], or to the deliberate inaccuracies and concealment in the reporting of COVID-19 infections, especially in lower-middle-income countries [5].

Given the high infectiousness and mortality of COVID-19, from the beginning of the pandemic, nations have tried to reduce the virus’ transmission through a range of measures, such as lockdowns (business and school closure; restricting interpersonal contact), social distancing, mask-wearing, hand hygiene, widespread testing of populations, and contact tracing [3,6,7,8,9,10,11,12,13,14]. In the first months of 2021, the prompt development of COVID-19 vaccines provided us with a new, effective way of dealing with the COVID-19 virus. The first authorized vaccine in Europe was the Pfizer-BioNTech COVID19 Vaccine, approved on 21 December 2020 by the European Medicines Agency [15], followed by others in subsequent months. By July 2022, 12,220,000,000 vaccine doses had been administered worldwide, covering almost 5,000,000,000 people with at least one dose [3]. Despite the differences between vaccine types (i.e., mRNA, viral vector, or inactivated virus vaccines; one or two shots), all the authorized vaccines showed a very high efficacy against the symptomatic COVID-19 infection (81% less after the last recommended dose) and hospitalization (84% less), with the prevention of peaks as high as 100 percent for the severe forms and lethal outcomes of the original SARS-CoV-2 [16,17]. A recent review showed that the COVID-19 vaccines on the market also have preventive effects against the new variants (i.e., Alpha, Beta, Gamma, and Delta), albeit in smaller percentages [17]. As for the Omicron variant, the preventive rates of commercial vaccines are still under investigation [17].

The mere existence and availability of rapid and functional vaccines, however, is not enough to avert or slow further waves of infection; it is necessary to vaccinate an appropriate number of subjects to establish a kind of herd immunity that can limit the contagions [18]. For this reason, the scientific community has put a great deal of effort into identifying possible psychological barriers against vaccination, such as implicit and explicit attitudes toward vaccination, vaccine hesitancy, readiness to change, risk perception, and mistrust or beliefs in conspiracy [18,19,20,21,22]. In particular, vaccine hesitancy and vaccine fear have emerged as some of the most important factors [21,23]. 

Well before the COVID-19 pandemic, vaccine hesitancy was defined as “a delay in accepting or refusing vaccination despite the availability of vaccination services, influenced by complacency, inconvenience in accessing vaccines, and a lack of confidence [24]. It was also listed as one of the top 10 threats to global health in 2019 by the World Health Organization [25]. The tendency and reasons for hesitancy may depend on a host of variables, such as the vaccines in question, as well as emotional, cultural, social, spiritual, and political factors [26,27]. Vaccine hesitancy, though already relevant, has played and continues to play a key role in the management of the COVID-19 pandemic [21]. 

Often, vaccine hesitancy goes hand in hand with vaccine fear. For instance, Diaz and colleagues found that the fear of adverse effects on fertility was the major cause of COVID-19 vaccine hesitancy in their sample of the American population [28]. Moreover, at this moment in history, information and even fake news (i.e., the harmless nature of COVID-19, poorly tested and experimental vaccines with hidden side effects such as infertility, DNA modification, and death) circulate freely on social media, influencing the general population’s opinion and fear about the COVID-19 vaccine and increasing their vaccine hesitancy [29,30]. 

### 1.1. How Long-COVID May Affect Vaccine Hesitancy

Long-standing, sickly body conditions due to chronic diseases or diseases that exhibit different symptoms over a long period of time [31] have an impact and affect people both mentally and physically. Contemporary examples of such impacts may be seen in patients affected by COVID-19, as the disease affects the human body over a long timeframe. Long-COVID-19 is a definition used to describe the disease in people who have recovered from the COVID-19 disease but still report lasting effects of the infection or have usual symptoms for much longer than expected [32]. Lately, there are a growing number of studies on long-COVID, whose symptoms can occur in many different ways [33,34,35,36,37].

The effects of these symptoms on the daily lives of individuals are also felt strongly. A study in the literature showed that only around 13% of the patients discharged from the hospital had their symptoms cleared, while the remainder still had some symptoms, even though the average period of 60 days had passed since their discharge [38]. These symptoms are wide-ranging and impact on human life, including fatigue, headaches, dyspnea, joint pain, chest pain, ageusia, anosmia, hair loss, and organ dysfunction in the lungs, heart, and brain [39,40,41,42]. However, contrary to popular belief, long-COVID symptoms may also emerge in patients who did not experience severe disease and hospitalization [43], and the number of people affected by this discomfort is increasing day by day. In addition to the physical consequences, people who experience the disease for a long time are also affected on a psychological and cognitive level. More specifically, long-COVID patients may have depressive and anxiety outcomes [42,44], post-traumatic stress disorder (PTSD) [45], persistent memory loss [46], and sleep problems [47] that may ultimately lead to weakened working capacity [46]. 

Memory is one of the core qualities of mankind, which separates us from other living creatures and made us strong during the evolutionary process. The experiences that make up our memory are our source of knowledge, which helps us to understand and interpret the variables of the environment and create our memories. Acquired knowledge blends with the variables to which we are exposed (emotions, environment, etc.) and creates our attitudes. The experience of events and facing of their consequences affect our approach to exact or similar events in the future [48]. Additionally, humans tend to question whether they are biased in regard to an action after they perform said action based on their previously acquired knowledge [49]. This questioning helps the individual to evaluate the results of their actions and the desirability of their actions on behalf of others. This orientates one’s future approach to said actions [50].

Psychological models can help us to understand how long-term COVID experiences may affect human psychology. First of all, long-term outcomes can constitute a negative experience in our memory that, according to “Self-perception theory” [51] and the “Biased-scanning hypothesis” [52], can lead to a change in behaviors and attitudes. Moreover, the vaccine hesitancy that people have is reasonably affected by this, because people who have experienced long-term COVID may want to avoid re-exposure to the physical and mental consequences they suffered. Fears, however, usually require much more effort in order to be modified [53] (see the systematic desensitization technique for phobias [54] and exposure and response prevention technique for obsessions [55]), thus creating a sort of contrasting relationship between people’s beliefs about vaccination derived from their fear of it and their past experience due to long-COVID. Based on the integration of the abovementioned theories, there could be a moment in time when people still fear vaccination but diminish their hesitancy, since one aspect is more malleable than the other. Eventually, the mental system will find an equilibrium, as envisaged by “Cognitive dissonance theory” [56], and the two components will be aligned again. 

### 1.2. Hypotheses Development

It is known that human attitudes are affected positively or negatively by various life experiences, and these attitudes affect human behavior [57,58]. At the same time, the effect of these attitudes on human life is a subject that has been addressed by the field of social sciences, dating back many years [59,60]. In the last couple of years, with the ongoing COVID-19 pandemic, the number of studies on vaccines and human attitudes has increased [61,62,63]. One of the prime reasons for this increase can be linked to vaccine hesitancy due to COVID in many countries [64,65,66]. Notably, people’s vaccine hesitancy was found to be associated with a plethora of personal and vicarious COVID-related experiences [67,68,69]. The authors of this study explored the possibility that long-lasting symptoms (i.e., long-COVID) may affect vaccine hesitancy levels, as was observed in the case of other COVID-related experiences. In this study, the researchers claimed that Bem’s (self-perception theory) and Albarracín’s (biased-scanning hypothesis) theories could provide an effective and clear way of explaining the relationship between COVID-19 status and vaccine hesitancy [51,52]. Since past experiences can affect the evaluation of new experiences, in some cases, past experiences are overlooked, while new experiences are prioritized. 

In our case, we hypothesized that long-COVID may be considered a negative experience able to affect (i.e., lessen) vaccine hesitancy, while simply contracting COVID is not enough to trigger this rearrangement process. Indeed, as in the case of influenza-like illnesses, being exposed to a “manageable” symptomatology is not enough to push people towards higher levels of influenza vaccination uptake [70]. In line with these expectations, the hypotheses of our study can be listed as follows:

**Hypothesis** **1** **(H1).** *People who have not had COVID have lower vaccine hesitancy than individuals who have had COVID, without developing long-lasting symptoms.*

**Hypothesis** **2** **(H2).** *People who have developed long-COVID symptoms have the same vaccine hesitancy levels as people who have not had COVID.*

**Hypothesis** **3** **(H3).** *People who have developed long-COVID symptoms have lower vaccine hesitancy levels than people who have had COVID but did not experience long-lasting symptoms.*

Consistent with the psychological models and evidence explained above, implying that fear is more resistant to change compared with other emotional and cognitive aspects [53,54,55], we can formulate a fourth hypothesis:

**Hypothesis** **4** **(H4).** *No difference in terms of vaccination fear is expected between our three clusters.*

Thus, people who did not have COVID-19, people who had COVID but did not develop long-lasting symptoms, and people who had COVID and developed long-COVID syndrome are expected to show no change in their vaccine fear as a result of their specific experiences.

## 2. Method

To test our hypotheses, we decided to rely on already gathered and published data [71] that were collected from 20 December 2021 to 10 January 2022, since the Italian government made COVID-19 vaccination mandatory on that date for people older than 50 years old [72], and this decision may have greatly affected vaccine hesitancy levels among this specific population, as well as others. Given that our goal was to investigate how COVID-19 experiences (i.e., no infection, infection without long-lasting symptoms, and long-COVID) affected vaccine hesitancy and vaccination fear levels in a situation of decision-making freedom and not legal obligation, we had to use already collected and analyzed data, acknowledging a possible type-1 error increase due to this decision. 

### 2.1. Participants

A total of 1111 Italian speakers recruited online participated in the study, including 26.6% men and 68.9% women, with a mean age of 38.33 (standard deviation = 13.94). Of these 1111 people, 28% reported having been infected by COVID-19, and 47% of people who had had COVID-19 also reported having experienced long-lasting symptoms (i.e., N = 149). Part of the sample had a high school educational level (38.3%) while 25.9% had a master’s degree. Most of the sample were coupled or married (67.5%), followed by single (25.7%), divorced or separate (5.5%), and widowed (1.4%). Regarding cohabitation, the vast majority were living with family members (50.3%) or partners (25.7%), with 12.9% living alone.

### 2.2. Procedure

The recruitment for this study was carried out through a message containing the survey link, which was distributed via the web, social media (Facebook, Instagram, LinkedIn), and mailing lists to all potential participants. A disclaimer form was included at the beginning of the survey to inform the participants of the aim of the study and the full anonymity regarding their answers. In order to continue with the questionnaires, each participant had to accept the terms of the study, which complied with the Helsinki declaration, Italian legal requirements of privacy and informed consent (Law Decree DL-101/2018), EU regulations (2016/699), and (APA) guidelines. The completion time for the survey was around 10 min. 

### 2.3. Measures

Fear of vaccination: The Fear of Vaccination Scale (VFS-6) [73] was used in its Italian-validated form, developed by Duradoni and colleagues [71]. The scale is composed of six items measured on a 5-point scale from 1 (strongly disagree) to 5 (strongly agree), with scores ranging from 6 to 30. The scale has two dimensions: cognitive and somatic. Higher scores in these dimensions reflect higher levels of fear related to vaccination. The scale showed optimal internal reliability (VFS-6_(cognitive)_ ω = 0.86; α = 0.85; VFS-6_(somatic)_ ω = 0.87; α = 0.84).

Vaccination hesitancy: The 5-C scale [74] measures the multidimensional conceptualization of vaccine hesitancy. The scale is composed of 15 items measured on a 5-point scale ranging from strongly disagree to strongly agree and encompasses 5 dimensions, namely confidence (i.e., trust in the effectiveness and safety of vaccines), complacency (i.e., vaccination not deemed as a necessary preventive action), constraints (i.e., reporting issues that prevent one from getting vaccinated), calculation (i.e., extensive engagement in evaluating the risks of infections and vaccination to derive a good decision), and collective responsibility (i.e., the willingness to protect others through one’s own vaccination). More specifically, the confidence and collective responsibility dimensions underline a positive attitude toward vaccination (+), while complacency and constraints represent a negative attitude toward vaccination (−). For the calculation dimension, no clear interpretation could be drawn, since it could highlight a higher hesitancy, but not necessarily (0). The item examples are as follows: confidence (“I am completely confident that vaccines are safe”), complacency (“Vaccine-preventable diseases are not so severe that I should get vaccinated”), constraints (“Everyday stress prevents me from getting vaccinated”), calculation (“For each and every vaccination, I closely consider whether it is useful for me”), and collective responsibility (“I get vaccinated because I can also protect people with a weaker immune system”). Cronbach’s α for the 5-C scale ranges from 0.67 to 0.88.

### 2.4. Statistical Analysis

Prior to the participants’ recruitment, efforts were made to identify an adequate sample size for this study. Since the authors planned to use one-way ANOVA and Welch’s *t*-tests (as a pairwise comparison) to assess vaccine hesitancy and vaccination fear differences, several power analyses were performed. We relied on G*Power software to accomplish this procedure [75,76]. For ANOVA, a sample of 246 was required to achieve a statistical power of 0.80, while assuming a small effect size (d = 0.20). However, since we expected a relatively severe imbalance between our clusters, we did our best to predict the adequate number of individuals in each category based on the most recent available data. The number of COVID-infected people in Italy rose from 6.8% in May 2021 [12] to around 10% in January 2022 [77]. Therefore, for the pairwise comparison (*t*-test family tests), 76 people that had been infected were required to enable us to detect a small–medium effect size (i.e., d = 0.30) and achieve a statistical power of 0.80. Eventually, since around half of the infected people develop long-COVID [31], we would have required 36 people with long-lasting symptoms to allow for a comparison able to capture medium differences (i.e., d = 0.50), while achieving the same power. Since the sample was composed of 1111 people, with a less severe imbalance than expected (28% infected people, of which 47% had long-lasting symptoms), our sample size appeared to be adequate, even to detect smaller effect sizes, compared with those revealed in the power analysis. 

## 3. Results

As a first step, we produced descriptive statistics disaggregated by condition (i.e., whether a person has been infected with COVID-19 or not and developed long-COVID symptoms) for all the continuous variables collected (Table 1).

We then proceeded to investigate the differences between the three groups based on the vaccination fear and vaccine hesitancy levels through one-way ANOVA analysis after checking for the assumptions (i.e., normality through asymmetry and kurtosis values, and homogeneity of variance). Table 2 summarizes the results obtained. 

As shown in Table 2, vaccination fear did not appear to be significantly different between groups, while four out of the five vaccine hesitancy dimensions appeared, on average, to be different. The only exception in line with our hypotheses was the calculation dimension. However, since we expected a non-linear modification of the vaccination fear and vaccine hesitancy scores between groups, we still conducted pairwise comparisons, including all the continuous variables to avoid excluding possible undetected effects in the ANOVA analysis. We performed such comparisons using Welch’s *t*-test to better address the issues related to the different sample sizes between groups (Table 3). For the vaccination fear, the pairwise comparisons were able to detect a statistically significant difference only in the somatic component between not-infected people and those with long-COVID. However, this difference appeared to be small in terms of the effect size. 

**Figure 1:**
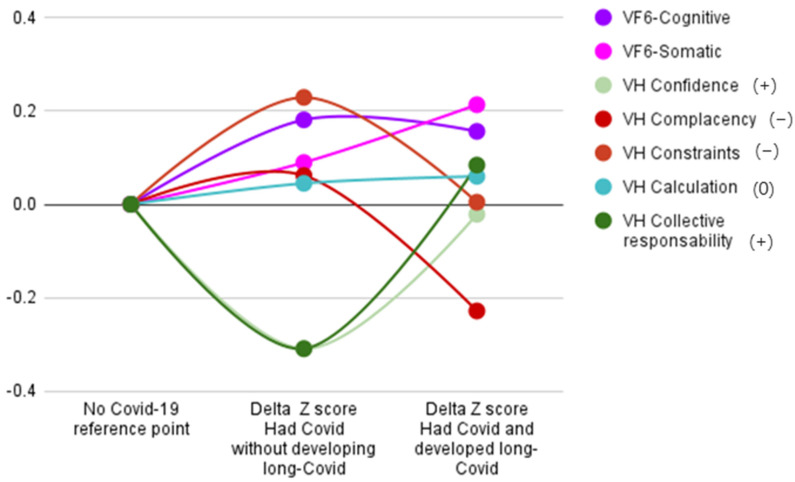
Standardized differences in vaccine hesitancy and vaccination fear between groups. Vaccine hesitancy (VH) dimensions marked with a plus symbol underline a positive opinion of vaccination, while VH dimensions marked with a minus sign are those related to a negative opinion of vaccination. Zero (0) was used for the calculation, since no clear interpretation could be drawn for that dimension.

To better visualize the obtained results, we produced a picture using the standardized mean differences for each dimension (Figure 1). People who had been infected with COVID-19 appeared to have, on average, higher levels of complacency and constraints and lower scores for confidence and collective responsibility compared to people whom the virus had not infected, thus highlighting the overall higher vaccine hesitancy of people who had been infected with COVID-19. As shown in Table 3, people who had experienced long-COVID syndrome, on average, showed decreased hesitancy compared to those who had been infected but did not experience long-lasting symptoms and, in general, they reported hesitancy scores similar to those obtained by never-infected people, with the sole exception of complacency, which turned out to be even lower. No statistically significant variation was observed between the groups for the calculation dimension, thus confirming the one-way ANOVA results. 

## 4. Discussion

Since 2019, the COVID-19 pandemic has affected hundreds of thousands of people, causing the death of more than 6,000,000 human beings all over the world [3]. For almost two years, the infections were managed only by asking citizens to stay at home, the use of facial masks, maintenance of interpersonal distance, and frequent disinfection of hands and surfaces [3,7,9,10]. Fortunately, in early 2021, specially created vaccines against COVID-19 were authorized and were able to reduce the likelihood of COVID-19 infection, severe symptoms, hospitalization, and lethal outcomes [16,17]. The literature also showed that, in terms of the likelihood of experiencing long-COVID, the vaccines were found to be effective, whether they are given before or after infection [78,79]. However, the mere presence of functional and authorized vaccines does not in itself ensure that people will agree to be inoculated [18]. Indeed, much effort has been spent, in recent times, on studying the barriers to vaccination, such as vaccine hesitancy and vaccine fear, so as to maximize the number of vaccinated people [19,21,23]. 

This study was carried out among individuals who had different self-reported COVID-19-related experiences (i.e., no infection, infection with a “normal” symptomatology, and long-COVID-19). The reported results appeared to be consistent with the two different psychological models that the authors integrated (i.e., self-perception theory [51] and the biased-scanning hypothesis [52]). Of course, the experience of individuals who had not contracted COVID-19 was not enough to change either their attitudes (i.e., vaccine hesitancy levels) or fears. The same was observed regarding people who had had mild symptoms. In this case, the magnitude of the negative experience did not seem to be enough to break the typical human tendency towards cognitive homeostasis, which included, for them, higher levels of hesitancy compared to people who had not had COVID (H1; [70]). The long-COVID experience appeared, instead, to be strong enough to ignite a modification in people’s psyches. People with long-COVID showed similar scores in terms of their vaccine hesitancy to people that had not had COVID (H2) and, therefore, showed a lower overall level of hesitancy than people who had had COVID without experiencing long-lasting symptoms (H3). Nonetheless, no sensible modification was observed regarding the vaccination fear scores (H4). Indeed, not all the components of the human psyche are expected to change at the same rate, due to their greater or lesser resistance [53,54,55]. In this sense, the interplay and relationship between vaccine hesitancy and vaccination fear should be considered dynamic. Therefore, in our study, it was possible to observe a contrasting/misaligned relationship between vaccine hesitancy (more prone to modification) and vaccination fear (less prone) that is expected to realign itself only at the end of a dynamic process.

Future studies should examine the effects of long-COVID-19 on attitudes in greater depth. The literature on COVID-19 and vaccine hesitancy research does not broadly address long-COVID-19 [33,34,35,36,37]. However, studies on people who have experienced COVID-19 or who have been exposed to the experiences of people close to them revealed that there is a significant relationship between vaccine hesitancy and these experiences [67,68,69]. Moreover, vaccine hesitancy and fear may be different before and after vaccination based on the side effects. Future research should consider this possibility and explore the robustness of our findings by comparing vaccinated and non-vaccinated participants. More in-depth analyses concerning gender, sex, and education should be performed as well, since these characteristics have been shown to affect vaccine hesitancy and fear levels [71,80]. 

Vaccine hesitancy is a complicated topic that places public health officials and doctors at risk of communication issues [27]. Given the COVID-19 pandemic, the vaccine hesitancy rate [64,81] remains dire, and the protection provided by regular vaccination against COVID-19 is underestimated. This situation has placed an obligation on the health authorities to understand the public’s attitude towards vaccines and take action in order to positively influence individuals’ thoughts about vaccines. Accordingly, it is believed that various information campaigns to be carried out in the future will create positive awareness about the vaccine fear and vaccine hesitancy status of the public. Moreover, the literature showed that identification with both membership groups, the national government and supranational entities (e.g., the European Union) can model and affect vaccine fear and vaccine hesitancy, helping to deal with the COVID-19 pandemic [82,83].

In addition, studies have shown the positive effects of psychological support provided to individuals who experience COVID or long-COVID symptoms [84,85]. Identifying people with these experiences through various surveys and helping them via a therapy center or help hotline could increase awareness in the individual context. However, when evaluating the findings, there are several limitations of the current study that must be emphasized.

Furthermore, studies in the literature have shown that with the emergence of the COVID-19 pandemic, the rate of individual use of social media has increased [86,87,88,89,90]. Many studies have also shown that individuals are affected by misinformation about vaccines on social media [91,92,93]. Another action plan that can be realized is to use social media to reach large audiences, which could effect a reduction in vaccine hesitancy among individuals. Social media is emerging as one of the most functional tools used to inform the population today [94,95]. A social media collaboration between government agencies and volunteers could create a snowball effect that will inform the large masses about vaccine hesitancy. Moreover, this social media campaign is also a good action plan for informing individuals about the symptoms of long-COVID-19 and reinforcing the importance of the vaccine.

First, we must state that causality cannot be inferred, because the research was a cross-sectional study, and there is no direct evidence of causality between the variables. Moreover, we measured the participants’ long-COVID-19 symptoms using the self-report technique; there were no medical diagnoses. Personal differences may affect the results of the self-report technique. Therefore, future studies based on medical diagnoses are necessarily needed. Lastly, given that the COVID-19 pandemic has been experienced with immense consequences in Italy [96], the fact that all the participants in the study were recruited in the Italian sample also raises different questions about generalization.

## 5. Conclusions

Overall, our work highlighted how the long-COVID experience may affect people’s vaccine hesitancy levels (by diminishing it), but not their vaccination fear. At the same time, people who had mild symptoms showed on average a higher vaccine hesitancy, which may put them more at risk of future infections.

## Figures and Tables

**Table 1:** Descriptive statistics.

	Had No COVID-19 (N = 795)		Had COVID without Developing Long-COVID (N = 167)		Had COVID-19 and Developed Long-COVID (N = 149)	
Variable	M	s.d.	M	s.d.	M	s.d.
VFS-6 cognitive	7.42	4.04	8.15	4.38	8.05	3.98
VFS-6 somatic	4.88	2.91	5.14	3.24	5.50	3.25
VH confidence	10.39	3.72	9.24	3.75	10.31	2.88
VH complacency	5.35	2.76	5.52	3.24	4.72	2.36
VH constraints	4.69	2.23	5.2	2.46	4.70	2.37
VH calculation	10.99	2.68	11.11	2.60	11.15	2.54
VH collective responsibility	12.05	2.98	11.13	3.29	12.3	2.63

Note: VFS-6 = Vaccination Fear Scale; VH = vaccine hesitancy; M = mean/average; s.d. = standard deviation.

**Table 2:** One-way ANOVA analyzing the differences between COVID-19-related conditions.

Criterion Variable	Mean Squares	d.f.	F	*p.*
VFS-6 cognitive	53.89	2; 1110	3.23	0.05
VFS-6 somatic	25.65	2; 1110	2.83	0.06
VH confidence	92.23	2; 1110	8.32	<0.001
VH complacency	138.73	2; 1110	17.82	<0.001
VH constraints	18.44	2; 1110	3.54	0.03
VH calculation	2.42	2; 1110	0.34	0.71
VH collective responsibility	70.16	2; 1110	7.88	<0.001

Note: VFS-6 = Vaccination Fear Scale; VH = vaccine hesitancy; F = Fisher F value; d.f. = degrees of freedom.

**Table 3:** Pairwise comparison through Welch’s *t*-test.

	Had No COVID-19 vs. Had COVID without Developing Long-COVID			Had No COVID-19 vs. Had COVID and Developed Long-COVID			Had COVID without Developing Long-COVID vs. Had COVID and Developed Long-COVID		
Criterion Variable	t	d.f.	d	t	d.f.	d	t	d.f.	d
VFS-6 cognitive	−1.99	229.25	n.c.	−1.79	209.13	n.c.	0.20	313.89	n.c.
VFS-6 somatic	−0.97	226.10	n.c.	−2.16 *	195.24	−0.20	−0.97	309.65	n.c.
VH confidence	3.70 ***	225.13	0.33	0.31	228.20	n.c.	−2.87 **	308.22	−0.32
VH complacency	−4.35 ***	219.61	−0.39	2.89 **	230.81	0.25	5.67 ***	302.43	0.64
VH constraints	−2.48 *	226.63	−0.22	−0.02	199.99	n.c.	1.86	312.15	n.c.
VH calculation	−0.55	245.40	n.c.	−0.74	241.59	n.c.	−0.16	311.60	n.c.
VH collective responsibility	3.36 **	226.67	0.29	−1.04	225.37	n.c.	−3.53 ***	310.33	−0.40

Note: VFS-6 = Vaccination Fear Scale; VH = vaccine hesitancy; t = Welch’s *t*-test value; d = Cohen’s d value; *** = *p* < 0.001; ** = *p* < 0.01; * = *p* < 0.05; d.f. = degrees of freedom; n.c. = not computed due to a not statistically significant result.

## Data Availability

The data presented in this study are available on request from the corresponding author.
